# 晚期肺腺癌EGFR-TKIs对后续培美曲赛化疗的影响

**DOI:** 10.3779/j.issn.1009-3419.2012.05.08

**Published:** 2012-05-20

**Authors:** 向迎 王, 友如 刘, 志强 高, 银玲 江, 宝惠 韩, 丽岩 姜

**Affiliations:** 200030 上海，上海交通大学附属胸科医院肺内科 Department of Pulmonary, Chest Hospital Affiliated to Shanghai Jiaotong University, Shanghai 200030, China

**Keywords:** 肺肿瘤, 培美曲赛, 表皮生长因子酪氨酸激酶抑制剂, Lung neoplasms, Pemetrexed, Epidermal growth factor receptor-tyrosine kinase inhibitors

## Abstract

**背景与目的:**

培美曲赛和表皮生长因子受体-酪氨酸激酶抑制剂（epidermal growth factor receptor-tyrosine kinase inhibitors, EGFR-TKIs）对于有*EGFR*基因突变的非小细胞肺癌（non-small cell lung cancer, NSCLC）患者能获得比野生型更好的疗效。本研究旨在通过临床观察肺腺癌患者EGFR-TKIs进展后后续培美曲赛化疗的疗效和毒副反应，分析EGFR-TKIs对后续培美曲赛化疗的影响。

**方法:**

收集Ⅲ期和Ⅳ期肺腺癌患者，根据有无EGFR-TKIs治疗史分为靶向治疗组和非靶向治疗组。所有患者接受培美曲赛（500 mg/m^2^），均为二线及二线以上治疗。按照RECIST（Response Evaluation Criteria in Solid Tumors）1.0标准评价培美曲赛疗效，NCI-CTC（National Cancer Institute Common Toxicity Criteria）4.0标准评价毒副反应。研究终点为疾病控制率（disease control rate, DCR）、无进展生存期（progression free survival, PFS）和总生存期（overall survival, OS）。

**结果:**

靶向治疗组57例和非靶向治疗组56例患者，DCR分别为77.2%和67.9%（*P*=0.367），中位PFS为5.95个月和3.55个月（*P*=0.535），中位OS为10.10个月和8.24个月（*P*=0.432），靶向治疗组优于非靶向治疗组，但两组差异无统计学意义。常见毒性反应为Ⅰ度-Ⅱ度血液学毒性和胃肠道反应。靶向治疗组有2例患者因不能耐受的毒副反应而中断培美曲赛治疗，非靶向治疗组无因毒副反应而停药者。靶向治疗组和非靶向治疗组各有1例患者因Ⅳ度骨髓抑制而减量；分别有5例和9例患者因主观因素治疗延迟，但无严重毒副反应。

**结论:**

EGFR-TKIs后续培美曲赛化疗的DCR、PFS和OS有改善趋势，但两组差异无统计学意义；培美曲赛序贯应用具有良好的安全性，可以作为EGFR-TKIs进展后的挽救性治疗。

## Abstract (en)

**Background and objective:**

Pemetrexed and epidermal growth factor receptor (EGFR)-tyrosine kinase inhibitors (TKIs) were used in patients with EGFR mutation to determine their effects. This study analyzed the influence of EGFR-TKIs on pemetrexed by observing the clinical efficacy and toxicity of pemetrexed following responses to EGFR-TKIs.

**Methods:**

Pulmonary adenocarcinoma patients were divided into EGFR-TKIs and no-EGFR-TKI groups according to the targeted therapy. All patients received pemetrexed (500 mg/m^2^) as second (or higher)-line treatment. The Response Evaluation Criteria in Solid Tumors (version 1.0) were used to evaluate the response to pemetrexed. Adverse events were classified based on version 4.0 of the National Cancer Institute Common Toxicity Criteria.

**Results:**

There were 57 patients in the EGFR-TKIs group and 56 in the no-EGFR-TKIs group. The disease control rates (DCRs) were 77.2% and 67.9% (*P*=0.367). The progression free survival (PFS) periods were 5.95 and 3.55 months (*P*=0.535). The overall survival (OS) periods were 10.10 and 8.24 months (*P*=0.432). However, these values were not statistically significant. The common toxicities of pemetrexed were hematologic and gastrointestinal (grades Ⅰ and Ⅱ). Two patients in the EGFR-TKIs group discontinued pemetrexed because of severe toxicities, which were not observed in the no-EGFR-TKIs group. Both groups had one patient who reduced dosage because of myelosuppression (grade Ⅳ). There were five and nine patients in the EGFR-TKIs and no-EGFR-TKIs groups, respectively, who delayed therapy not because of severe toxicities but due to subjective factors.

**Conclusion:**

The DCRs, PFS periods, and OS periods of the patients administered with pemetrexed following EGFR-TKIs were better than those of the EGFR-TKIs group, but the differences were not statistically significant. Therefore, sequential pemetrexed administration caused negligible toxicities and can be used in adenocarcinoma therapy following responses to EGFR-TKIs.

目前肺癌是全球癌症死亡的首要原因，超过乳腺癌、结直肠癌、前列腺癌的总和^[1]^。非小细胞肺癌（non-small cell lung cancer, NSCLC）约占80%以上，其中，非鳞癌患者约77%，包括腺癌、大细胞癌等^[2]^。

表皮生长因子受体（epidermal growth factor receptor, EGFR）在恶性肿瘤发生发展及肿瘤血管形成中起着十分重要的作用，是近年来肿瘤治疗及靶向药物研究的热点。EGFR酪氨酸激酶抑制剂（EGFR tyrosine kinase inhibitors, EGFR-TKIs）目前多用于NSCLC患者一线或者二线治疗中。一项*meta*分析结果^[3]^表明，有*EGFR*基因突变的晚期NSCLC患者一线EGFR-TKIs治疗可以取得优于化疗的有效率、无进展生存期（progression free survival, PFS）和生活质量，而EGFR基因突变多见于腺癌患者^[4]^。

培美曲赛是一种新型抗叶酸代谢细胞毒药物，能竞争性抑制胸腺嘧啶合成酶（TS）、二氢叶酸还原酶（DHFR）及甘氨酰胺核苷酸甲基转移酶（GARFD）等叶酸依赖性酶，造成叶酸代谢和核苷酸合成过程的异常，从而抑制肿瘤细胞的生长。由于低毒性及良好的耐受性，培美曲赛广泛用于非鳞肺癌的一线、二线及维持治疗^[5, 6]^。研究^[7]^表明，培美曲赛用于NSCLC的有效率为8.3%，有*EGFR*基因19和21位点突变的肺癌患者能获得比野生型更好的有效率（12.9% *vs* 1.6%, *P*=0.016）及PFS（3.9个月*vs* 2.3个月，*P*=0.030）。而靶向治疗进展后*EGFR*基因耐药突变的出现对培美曲赛的影响尚不清楚，相关研究较少。本研究通过临床观察复治的Ⅲ期和Ⅳ期肺腺癌患者EGFR-TKIs进展后后续培美曲赛化疗的疗效和毒副反应，回顾性分析EGFR-TKIs进展后对后续培美曲赛治疗的影响。

## 1 资料与方法

### 1.1 资料

2007年7月-2011年7月在上海市胸科医院住院的Ⅲ期和Ⅳ期肺癌患者，细胞学或者病理学确诊为肺腺癌，PS评分0分-2分，接受过至少1个疗程培美曲赛二线及二线以上化疗，培美曲赛（力比泰，Alimta）剂量为500 mg/m^2^，口服地塞米松和肌肉注射VitB_12_预处理，每天口服补充叶酸1, 000 μg，直至中断培美曲赛化疗后3周。每3周-4周为1个治疗周期。通过收集患者住院期间病史资料获取临床观察研究资料，电话随访患者的生存资料。

### 1.2 方法

本研究观察的首要终点为PFS，次要终点为疾病控制率（disease control rate, DCR）、总生存期（overall survival, OS）。DCR包括CR+PR+SD。按照RECIST 1.0标准每个周期评价疗效，分为完全缓解（complete response, CR）、部分缓解（partial response, PR）、疾病稳定（stable disease, SD）、疾病进展（progressive disease, PD）。PFS是指培美曲赛首次化疗直至发现疾病进展或者研究随访终止的时间。OS是指培美曲赛首次化疗直至患者任何原因的死亡或者研究随访终止的时间。根据NCI-CTC 4.0标准评价培美曲赛的毒副反应。

### 1.3 统计学方法

用SPSS 16.0软件包进行数据统计学分析。两组基线特征及疗效的比较用*Pearson* χ^2^检验，中位PFS和OS生存分析采用*Kaplan-Meier*法。以*P* < 0.05为差异有统计学意义。

## 2 结果

### 2.1 结果

本研究共入组113例患者，其中，57例既往曾口服EGFR-TKIs靶向药物，56例既往未采用EGFR-TKIs治疗。两组患者基本特征无统计学差异，具体见表 1。靶向治疗组中，一线靶向治疗者2例，既往口服吉非替尼患者34例，厄洛替尼患者20例，吉非替尼及厄洛替尼均服用过者3例。随访结束时，靶向治疗组有21人存活，非靶向治疗组有30人存活。靶向治疗组和非靶向治疗组PR患者所占比例分别为5.3%和1.8%，SD患者所占比例分别为71.9%和66.1%，DCR分别为77.2%和67.9%，两组差异无统计学意义（*P*=0.367）。靶向治疗组和非靶向治疗组中位PFS分别为5.95个月和3.55个月，总体中位PFS为4.40个月，两组差异无统计学意义（*P*=0.535）。靶向治疗组和非靶向治疗组中位OS分别为10.10个月和8.24个月，总体中位OS为9.00个月，两组差异无统计学意义（*P*=0.432）（图 1，图 2）。

**1:** 患者基本特征 Baseline characteristics of patients

Variable	With EGFR-TKIs group	Without EGFR-TKIs group	*P*
Gender			0.300
Male	26	31	
Female	31	25	
Smoking			0.274
Yes	14	19	
No	43	37	
Response of chemotherapy			0.128
DCR	48	54	
PD	6	2	
Stage			0.124
Ⅲa	8	2	
Ⅲb	7	10	
Ⅳ	42	44	
Operation			0.333
Yes	10	14	
No	47	42	
Pemetrexed			0.508
Platinum	39	35	
No-Platinum	18	21	
Age (58, 31-77)			0.634
< 65	42	39	
≥65	15	17	
ECOG PS			0.354
0-1	54	54	
2	3	2	
Chemotherapy regimen			0.074
Platinum	51	56	
No-platinum	3	0	
Number of prior therapies			0.392
1	3	7	
2	32	30	
> 2	22	19	
Radiotherapy			0.901
Yes	21	20	
No	36	36	
EGFR-TKIs: epidermal growth factor receptor-tyrosine kinase inhibitors; DCR: disease control rate; PD: progressive disease; PS: performance status.			

**1:**
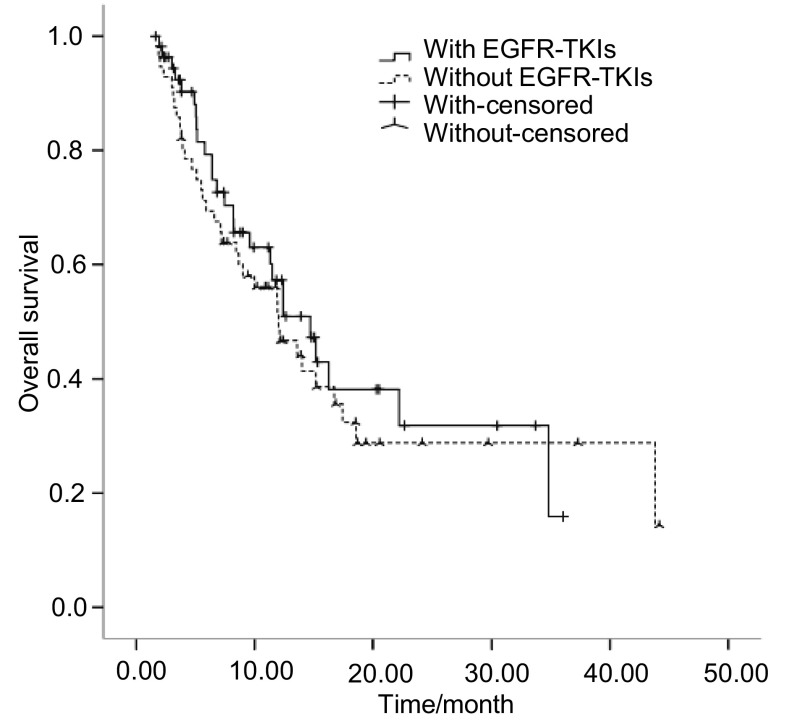
总生存期（*Kaplan-Meier*曲线） Overall survival (*Kaplan-Meier* curves) in with or without EGFR-TKIs group

**2:**
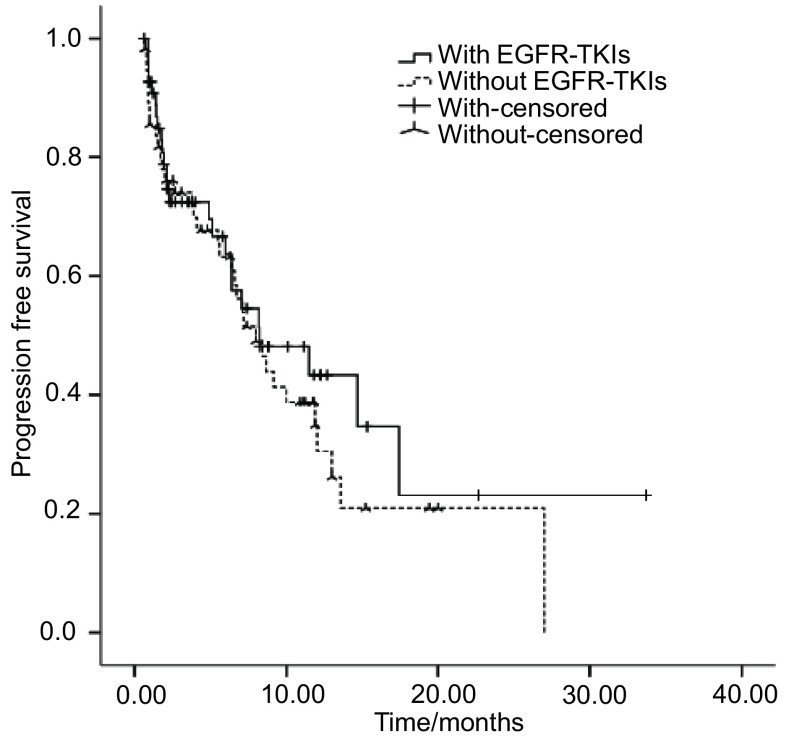
无进展生存时间（*Kaplan-Meier*曲线） Progression free survival (*Kaplan-Meier* curves) in with or without EGFR-TKIs group

### 2.2 毒性反应

最常见的毒性反应为Ⅰ度-Ⅱ度血液学毒性和胃肠道反应，具体见表 2。其它较少见毒性反应靶向治疗组和非靶向治疗组分别为：皮疹（6例和3例，Ⅰ度-Ⅲ度），乏力（2例和1例，Ⅰ度-Ⅲ度）。另外，靶向治疗组可见肝功能损害（2例，Ⅰ度-Ⅱ度），周围神经毒性（2例，Ⅰ度-Ⅱ度）。靶向治疗组有2例患者因不能耐受的毒副反应而中断培美曲赛治疗，1例皮疹（Ⅲ度），1例乏力（Ⅲ度）；非靶向治疗组无因毒副反应而停药者。靶向治疗组和非靶向治疗组各有1例患者因Ⅳ度骨髓抑制而减量，分别为贫血和白细胞减少。靶向治疗组和非靶向治疗组分别有5例和9例患者因主观因素延迟治疗，但无严重毒副反应。本研究中，靶向治疗组血红蛋白减少（*P*=0.020）和血小板减少（*P*=0.029）较非靶向治疗组多，具有统计学差异。

**2:** 常见毒副反应 Toxicities

Toxicity	With EGFR-TKIs group	Without EGFR-TKIs group	*P*
Leucopenia			0.193
Ⅰ	10	5	
Ⅱ	3	4	
Ⅲ	3	0	
Ⅳ	0	1	
Thrombocytopenia			0.029
Ⅰ	3	0	
Ⅱ	0	4	
Ⅲ	2	0	
Ⅳ	0	0	
Anemia			0.020
Ⅰ	9	0	
Ⅱ	1	1	
Ⅲ	0	1	
Ⅳ	1	0	
Abnormal gastrointestinal			0.415
Ⅰ	5	9	
Ⅱ	5	2	
Ⅲ	1	2	
Ⅳ	0	0	

## 3 讨论

培美曲赛是多靶点叶酸抑制剂，其中胸苷酸合酶（TS）的表达最重要。研究^[9-11]^表明，TS表达增加的患者培美曲赛疗效较差，TS表达水平已经成为NSCLC患者培美曲赛疗效的一个预测因子。TS的表达与临床病理类型相关，肺腺癌患者表达水平较鳞癌低，所以培美曲赛用于肺腺癌患者可以获得比鳞癌更好的疗效。Bepler等^[12]^研究发现，TS表达水平在不同的肺癌细胞株中有所不同，与*EGFR*野生型相比，有突变的H1650细胞株表达低水平的TS。研究^[8]^表明EGFR突变阳性的患者EGFR-TKIs治疗具有反应率和无进展生存期的优势。由此可见，TS表达和*EGFR*突变或许具有一定的相关性，而EGFR-TKIs治疗进展后*EGFR*基因耐药突变的出现是否会引起TS表达的改变从而影响培美曲赛疗效尚不清楚，目前尚未见到相关研究。

本研究通过临床观察Ⅲ期和Ⅳ期肺腺癌患者EGFR-TKIs进展后后续培美曲赛化疗的疗效和毒副反应，回顾性分析EGFR-TKIs对后续培美曲赛化疗的影响。本研究中，EGFR-TKIs靶向治疗的DCR达到92.7%，中位PFS为3.7个月，且所选取均为腺癌、亚裔人群，临床推断所选择人群*EGFR*突变率较高。

本研究表明，总体DCR为73.7%，中位PFS为4.40个月，中位OS为9.00个月，均优于NSCLC培美曲赛单药二线治疗的研究^[13]^。可能与以下两个方面有关：第一，本研究选择的均为肺腺癌患者。研究^[14]^表明，培美曲赛用于非鳞癌患者可以获得优于多西他赛的PFS（HR=0.82; 95%CI: 0.66-1.02; *P*=0.076）和OS（HR=0.78; 95%CI: 0.61-1.00; *P*=0.047），而鳞癌患者则劣于多西他赛。第二，本组患者人群均为亚裔，亚裔人群具有比欧美人群更高的*EGFR*突变率（30%和17%），相应的TS表达水平较低，培美曲赛可以获得更好的疗效。靶向治疗组和非靶向治疗组培美曲赛DCR分别为77.2%和67.9%，中位PFS分别为5.95个月和3.55个月，中位OS分别为10.10个月和8.24个月，两组DCR（*P*=0.367）、PFS（*P*=0.535）和OS（*P*=0.432）差异均无统计学意义，与Lee等^[15]^结论一致。可以看出，EGFR-TKIs进展后*EGFR*突变状态的改变对培美曲赛疗效有一定的影响，但没有统计学差异，尚需扩大样本量且进行前瞻性研究。

毒副反应方面，靶向治疗组和非靶向治疗组培美曲赛毒副反应大部分为Ⅰ度-Ⅱ度，耐受性好。另外，还有一些少见的毒性反应：靶向治疗组和非靶向治疗组分别为：皮疹（6例和3例，Ⅰ度-Ⅲ度）、乏力（2例和1例，Ⅰ度-Ⅲ度）。靶向治疗组可见肝功能损害（2例，Ⅰ度-Ⅱ度）、周围神经毒性（2例，Ⅰ度-Ⅱ度）。研究^[16]^关于培美曲赛和多西他赛无Ⅲ度-Ⅳ度毒性反应生存分析表明，培美曲赛具有比多西他赛更长的无Ⅲ度-Ⅳ度毒性反应生存时间（HR=0.60; 95%CI: 0.50-0.72; *P* < 0.0001）。本研究发现靶向治疗组血红蛋白减少（*P*=0.020）和血小板减少（*P*=0.029）较非靶向治疗组多，差异具有统计学意义。EGFR-TKIs骨髓抑制作用不明显，可能与本研究病例数较少有关。另外，本研究靶向治疗组有2例患者因不能耐受的毒副反应而中断培美曲赛治疗，1例皮疹（Ⅲ度），1例乏力（Ⅲ度），考虑可能与靶向药物的毒副反应累积有关。非靶向治疗组无因毒副反应而停药者。靶向治疗组和非靶向治疗组各有1例患者因Ⅳ度骨髓抑制而减量，分别为贫血和白细胞减少。

由于本研究均为Ⅲ期和Ⅳ期NSCLC患者，大多数患者的病理诊断是基于细胞学（如痰细胞学、胸水细胞学、穿刺细胞学或者支气管镜病理细胞学），肿瘤细胞少，目前尚缺乏统一的EGFR基因检测标准。而且，本研究为回顾性资料，所选多为4年前病例，故未行*EGFR*基因突变检测明确突变状态，仅对患者的临床因素进行分析。

综上所述，既往EGFR-TKIs使用，对后续培美曲赛化疗的DCR、PFS、OS和安全性有改善趋势，但无明显差异，是否能增强其疗效有待进一步研究，培美曲赛可以作为EGFR-TKIs进展后的挽救性治疗。
